# Impact of the COVID-19 pandemic on breast cancer referrals and diagnoses in 2020 and 2021: population-based study in England

**DOI:** 10.1093/bjs/znab426

**Published:** 2021-12-14

**Authors:** Toral Gathani, Gillian Reeves, David Dodwell, Kieran Horgan, Olive Kearins, Sau Wan Kan, Sian Sweetland

**Affiliations:** Cancer Epidemiology Unit, Nuffield Department of Population Health, Oxford, UK; Oxford University Hospitals NHS Foundation Trust, Oxford, UK; Cancer Epidemiology Unit, Nuffield Department of Population Health, Oxford, UK; Oxford University Hospitals NHS Foundation Trust, Oxford, UK; Clinical Trials Service Unit, Nuffield Department of Population Health, Oxford, UK; Department of Breast Surgery, St James’ Hospital, Leeds, UK; Breast Cancer Screening Programme, Screening Division, Public Health England, Birmingham, UK; Cancer Epidemiology Unit, Nuffield Department of Population Health, Oxford, UK; Cancer Epidemiology Unit, Nuffield Department of Population Health, Oxford, UK

##  

*Dear Editor*

Significant concerns have been raised about the impact of measures taken to manage the COVID-19 pandemic on the National Health Service (NHS) cancer services, including pausing of national screening programmes^1^. Data on cancer waiting times have shown the effect of the pandemic on breast cancer service referral and treatment activity in the first half of 2020^2^. By using routinely available national data for England^3^, breast cancer service referral and treatment activity in 2020 and 2021, compared with those in 2019, are reported here. Where data are available, urgent referral and treatment activity in England by age, ethnicity, and deprivation are reported^4^.

Data were extracted from two publicly available English data sets: the Cancer Waiting Times (CWT) data set^3^ and the COVID-19 Cancer Equity Data Packs^4^. The CWT data set reports monthly counts of urgent and routine breast referrals from primary to secondary care, and monthly counts of first treatments for breast cancer. The first treatment counts are a surrogate measure of the number of new diagnoses and include invasive (ICD-10 C50) and non-invasive (ICD-10 D05) breast cancer from any route to diagnosis, including routine screening which accounts for approximately 40 per cent of breast cancer diagnoses.

For referral and treatment data from the CWT data set, the monthly counts were grouped into five half-year time periods from January 2019 to June 2021, and present half-year counts and the ratio of half-year counts in 2020 and 2021 to the corresponding half-year counts in 2019 (*Table 1*). The COVID-19 Cancer Equity Data Packs provide additional information by age, ethnicity, and deprivation for urgent referrals and first treatment counts (see *Tables S1* and *S2*).

**Table 1:** Number of urgent referrals, routine referrals, and first treatments for breast cancer in 2019, 2020, and 2021 in England from Cancer Waiting Times data

	2019 January–June (*n*)	2020 January–June (*n*)	Ratio of counts January–June 2020 to 2019 (%)	2019 July–December (*n*)	2020 July–December (*n*)	Ratio of counts July–December 2020 to 2019 (%)	2019 January–June (*n*)	2021 January–June (*n*)	Ratio of counts January–June 2021 to 2019 (%)
**Urgent referral**	225 842	173 792	77.0	217 313	231 474	106.5	225 842	248 881	110.2
**Routine referral**	97 020	57 973	59.8	88 223	70 092	79.4	97 020	81 615	84.1
**First treatment**	23 867	19 965	83.7	24 588	19 844	80.7	23 867	23 142	97.0

Assuming 2019 represents a normal year for comparison, in the first half of 2020, there were 33 per cent fewer urgent, and 40 per cent fewer routine, referrals; however, in the second half of 2020, there were 7 per cent more urgent referrals and 20 per cent fewer routine referrals. During the first half of 2021, the volume of urgent referrals was 10 per cent higher and the volume of routine referrals remained 16 per cent lower, compared with 2019. Examination of monthly counts showed that urgent referral activity returned to usual levels by August 2020, with similar rates of recovery observed in the age, ethnicity, and deprivation groups examined (see *Table S1*).

In comparison to corresponding time periods in 2019, there were 16 per cent fewer first treatments for breast cancer in the first half of 2020, compared with 19 per cent fewer in the second half of 2020, but only 3 per cent fewer in the first half of 2021. The monthly number of first treatments largely recovered by December 2020, and recovery was slowest in people aged 50–69 years, the age range for which routine population-based screening is offered^5^ (see *Table S2*). The reduction in the number of first treatments suggests that there may be approximately 9500 ‘missing’ breast cancer diagnoses since the start of 2020 attributable to the pandemic. Roughly half of these are likely to be due to reduced screening activity from March to September 2020, and the remainder due to a reduction in the number of referrals.

Many uncertainties concerning breast cancer care existed in March 2020, and the measures introduced as a consequence of the pandemic undoubtedly had a significant impact. It is reassuring to observe that the recovery has been fairly rapid and subsequent lockdowns in November 2020 and January 2021 have had little impact on referrals and first treatment activity. Clinical teams have worked hard under challenging circumstances to ensure that breast cancer services can withstand COVID-19 surges. The impact of the pandemic on breast cancer services will continue to need further research, to better understand the long-term implications for patients overall and specific subgroups.

## Funding

T.G., S.W.K., and S.S. are funded, in part, by Cancer Research UK (C16077/A29186). D.D. is funded, in part, by Cancer Research UK (C8225/A21133). T.G., K.H., and D.D. are employees of NHS Trusts. O.K. is an employee of Public Health England.

*Disclosure*. The authors declare no conflicts of interest.

## Supplementary material

Supplementary material is available at *BJS* online.

## Supplementary Material

znab426_Supplementary_Data
