# Supplementary material for: Impact of the COVID-19 pandemic on breast cancer referrals and diagnoses in 2020 and 2021: population-based study in England
Source: Br J Surg. 2021 Dec 14;109(2):e29–30. doi: 10.1093/bjs/znab426 (PMC8807144; doi:10.1093/bjs/znab426)
Supplement: znab426_Supplementary_Data [file znab426_supplementary_data.docx]

**Supplementary material**

**Table S1: Ratio of urgent referral counts by age, ethnicity and deprivation from the Covid-19 Cancer Equity Packs**

| **(a) Age** | **Ratio of counts (95%CI) comparing 2021/20 to 2019 (%)** | | |
| --- | --- | --- | --- |
|  | **0-49** | **50-69** | **70+** |
| Jan-20 | 100.5 (98.5, 102.5) | 103.1 (100.2, 106.0) | 106.4 (102.0, 110.9) |
| Feb-20 | 94.9 (93.1, 96.7) | 93.8 (91.3, 96.3) | 97.6 (93.8, 101.6) |
| Mar-20 | 75.2 (73.8, 76.7) | 79.8 (77.6, 82.0) | 80.2 (76.9, 83.5) |
| Apr-20 | 45.5 (44.5, 46.6) | 43.0 (41.6, 44.4) | 36.8 (34.9, 38.8) |
| May-20 | 65.4 (64.1, 66.8) | 66.6 (64.6, 68.6) | 62.5 (59.8, 65.4) |
| Jun-20 | 77.2 (75.6, 78.7) | 80.0 (77.8, 82.2) | 75.5 (72.4, 78.8) |
| Jul-20 | 86.1 (84.4, 87.9) | 90.2 (87.8, 92.8) | 87.4 (83.9, 91.0) |
| Aug-20 | 102.3 (100.2, 104.4) | 107.7 (104.7, 110.7) | 95.9 (92.0, 99.9) |
| Sep-20 | 104.4 (102.4, 106.5) | 112.6 (109.5, 115.7) | 107.9 (103.6, 112.4) |
| Oct-20 | 105.8 (103.8, 107.8) | 112.1 (109.2, 115.1) | 104.9 (100.8, 109.1) |
| Nov-20 | 116.7 (114.6, 118.8) | 125.8 (122.6, 129.1) | 121.2 (116.6, 126.0) |
| Dec-20 | 113.0 (110.9, 115.1) | 118.0 (115.0, 121.1) | 112.7 (108.3, 117.3) |
| Jan-21 | 115.3 (113.2, 117.5) | 121.0 (117.8, 124.3) | 108.4 (104.0,113.1) |
| Feb-21 | 106.6 (104.6, 108.6) | 103.7 (101.0, 106.4) | 92.7 (89.0, 96.6) |
| Mar-21 | 104.9 (103.0, 106.7) | 108.7 (106.0, 111.4) | 108.7 (104.7, 113.0) |
| Apr-21 | 109.5 (107.5, 111.4) | 109.4 (106.7, 112.1) | 107.2 (103.1, 111.3) |
| May-21 | 117.1 (115.0, 119.2) | 115.5 (112.6, 118.5) | 114.3 (110.0, 118.8) |
| Jun-21 | 110.3 (108.3, 112.4) | 106.3 (103.6, 109.1) | 103.1 (99.2, 107.2) |
|  |  |  |  |
| **(b) Ethnicity** | **Ratio of counts (95%CI) comparing 2021/20 to 2019 (%)** | | |
|  | **White** | **Asian** | **Black** |
| Jan-20 | 98.8 (97.1, 100.5) | 113.4 (106.4, 120.8) | 112.2 (103.6, 121.5) |
| Feb-20 | 91.9 (90.3, 93.4) | 100.7 (94.6, 107.3) | 108.8 (100.6, 117.6) |
| Mar-20 | 75.8 (74.5, 77.1) | 73.6 (68.9, 78.6) | 81.5 (75.2, 88.4) |
| Apr-20 | 44.5 (43.6, 45.4) | 36.2 (33.4, 39.2) | 39.4 (35.7, 43.4) |
| May-20 | 67.8 (66.5, 69.0) | 50.2 (46.5, 54.2) | 49.1 (44.7, 53.8) |
| Jun-20 | 79.9 (78.5, 81.4) | 66.2 (61.8, 70.9) | 65.7 (60.5, 71.4) |
| Jul-20 | 90.3 (88.7, 91.9) | 69.5 (65.0, 74.3) | 78.1 (72.1, 84.7) |
| Aug-20 | 104.8 (102.9, 106.6) | 98.4 (92.1, 105.1) | 101.2 (93.6, 109.5) |
| Sep-20 | 109.5 (107.7, 111.5) | 88.4 (82.9, 94.4) | 103.3 (95.7, 111.5) |
| Oct-20 | 108.6 (106.8, 110.5) | 100.0 (93.9, 106.5) | 109.9 (101.7, 118.8) |
| Nov-20 | 122.4 (120.4, 124.3) | 101.5 (95.5, 108.0) | 124.7 (116.0, 134.1) |
| Dec-20 | 116.6 (114.8, 118.6) | 97.4 (91.6, 103.6) | 125.7 (116.5, 135.6) |
| Jan-21 | 118.0 (116.0, 120.0) | 100.4 (94.0, 107.1) | 111.3 (102.8, 120.5) |
| Feb-21 | 102.4 (100.8, 104.1) | 102.4 (96.2, 109.1) | 111.3 (103.0, 120.3) |
| Mar-21 | 104.7 (103.1, 106.4) | 99.8 (94.0, 106.0) | 110.6 (102.6, 119.2) |
| Apr-21 | 109.4 (107.7, 111.1) | 91.3 (86.0, 96.9) | 104.2 (96.9, 112.1) |
| May-21 | 114.9 (113.1, 116.7) | 109.4 (102.9, 116.4) | 110.1 (102.3, 118.5) |
| Jun-21 | 107.0 (105.3, 108.8) | 109.6 (103.2, 116.4) | 102.6 (95.3, 110.3) |
|  |  |  |  |
| **(c) Deprivation** | **Ratio of counts (95%CI) comparing 2021/20 to 2019 (%)** | | |
|  | **Least** | **Most** |  |
| Jan-20 | 103.0 (99.6, 106.5) | 99.8 (96.4, 103.3) |  |
| Feb-20 | 90.8 (87.9, 93.8) | 96.6 (93.4, 99.8) |  |
| Mar-20 | 75.7 (73.2, 78.2) | 78.6 (76.0, 81.3) |  |
| Apr-20 | 44.4 (42.7, 46.1) | 42.7 (41.0, 44.5) |  |
| May-20 | 69.7 (67.3, 72.2) | 63.6 (61.3, 66.0) |  |
| Jun-20 | 80.5 (77.9, 83.3) | 74.3 (71.7, 77.0) |  |
| Jul-20 | 90.0 (87.0, 93.1) | 84.1 (81.2, 87.1) |  |
| Aug-20 | 109.1 (105.4, 112.9) | 99.3 (95.9, 102.9) |  |
| Sep-20 | 110.4 (106.7, 114.1) | 101.4 (98.0, 105.0) |  |
| Oct-20 | 113.4 (109.7, 117.1) | 103.2 (99.9, 106.7) |  |
| Nov-20 | 127.0 (123.2, 131.0) | 113.4 (109.8, 117.1) |  |
| Dec-20 | 116.4 (112.8, 120.1) | 110.9 (107.3, 114.6) |  |
| Jan-21 | 120.7 (116.8, 124.6) | 114.5 (110.7, 118.4) |  |
| Feb-21 | 99.8 (96.7, 103.0) | 110.4 (106.9, 114.0) |  |
| Mar-21 | 107.7 (104.5, 111.1) | 108.8 (105.5, 112.3) |  |
| Apr-21 | 112.2 (108.9, 115.6) | 106.5 (103.2, 109.9) |  |
| May-21 | 119.9 (116.3, 123.7) | 110.8 (107.4, 114.4) |  |
| Jun-21 | 110.2 (106.8, 113.6) | 113.0 (109.4, 116.7) |  |

**Table S2: Ratio of first treatment counts by age, ethnicity and deprivation from the Covid-19 Cancer Equity Packs**

| **(a) Age** | **Ratio of counts (95%CI) comparing 2021/20 to 2019 (%)** | | |
| --- | --- | --- | --- |
|  | **0-49** | **50-69** | **70+** |
| Jan-20 | 98.2 (88.1, 109.3) | 99.7 (93.7, 106.1) | 98.9 (91.7, 106.7) |
| Feb-20 | 97.6 (87.7, 108.8) | 102.7 (96.2, 109.6) | 102.4 (94.8, 110.6) |
| Mar-20 | 101.6 (91.7, 112.4) | 125.2 (117.9, 133.1) | 134.7 (125.2, 144.8) |
| Apr-20 | 95.8 (86.2, 106.5) | 85.7 (80.4, 91.3) | 61.4 (56.5, 66.7) |
| May-20 | 71.2 (63.4, 80.0) | 48.5 (44.9, 52.3) | 46.0 (42.0, 50.4) |
| Jun-20 | 62.3 (55.3, 70.1) | 38.5 (35.4, 41.8) | 55.0 (50.4, 59.9) |
| Jul-20 | 74.6 (66.4, 84.0) | 46.8 (43.3, 50.7) | 74.1 (68.4, 80.2) |
| Aug-20 | 84.7 (75.6, 94.9) | 48.5 (44.9, 52.3) | 82.2 (76.1, 88.8) |
| Sep-20 | 97.6 (87.3, 109.0) | 68.6 (64.0, 73.5) | 87.7 (81.3, 94.6) |
| Oct-20 | 95.7 (85.7, 106.7) | 78.3 (73.1, 83.7) | 85.7 (79.3, 92.6) |
| Nov-20 | 100.0 (89.5, 111.8) | 89.7 (84.2, 95.7) | 94.3 (87.4, 101.8) |
| Dec-20 | 109.3 (98.2, 121.7) | 99.8 (93.8, 106.2) | 101.0 (93.6, 109.1) |
| Jan-21 | 87.8 (78.6, 98.1) | 95.5 (89.7, 101.7) | 84.6 (78.2, 91.6) |
| Feb-21 | 94.2 (84.5, 105.0) | 104.2 (97.7, 111.2) | 87.4 (80.6, 94.8) |
| Mar-21 | 81.9 (73.5, 91.2) | 101.0 (94.7, 107.7) | 93.2 (86.1, 100.8) |
| Apr-21 | 82.8 (74.1, 92.4) | 101.3 (95.3, 107.8) | 94.4 (87.7, 101.7) |
| May-21 | 94.3 (84.7, 105.0) | 105.8 (99.5, 112.4) | 99.1 (92.2, 106.6) |
| Jun-21 | 88.9 (79.9, 98.9) | 106.5 (100.3, 113.2) | 97.7 (90.8, 105.1) |
|  |  |  |  |
|  |  |  |  |
| **(b) Ethnicity** | **Ratio of counts (95%CI) comparing 2021/20 to 2019 (%)** | | |
|  | **White** | **Asian** | **Black** |
| Jan-20 | 98.2 (93.6, 103.0) | 97.5 (78.0, 122.0) | 123.0 (92.1, 164.3) |
| Feb-20 | 99.3 (94.5, 104.5) | 125.7 (101.2, 156.1) | 117.9 (87.9, 158.3) |
| Mar-20 | 121.6 (116.0, 127.4) | 135.2 (109.0, 167.7) | 146.2 (109.8, 194.6) |
| Apr-20 | 76.2 (72.4, 80.1) | 85.4 (67.5, 108.0) | 96.5 (71.7, 129.7) |
| May-20 | 50.3 (47.5, 53.3) | 61.2 (47.2, 79.3) | 75.6 (53.7, 106.5) |
| Jun-20 | 48.2 (45.4, 51.1) | 47.2 (35.9, 62.1) | 61.3 (43.9, 85.7) |
| Jul-20 | 60.3 (57.0, 63.7) | 69.9 (54.5, 89.6) | 50.5 (35.6, 71.6) |
| Aug-20 | 65.9 (62.4, 69.5) | 62.4 (49.5, 78.8) | 56.1 (40.6, 77.4) |
| Sep-20 | 78.3 (74.4, 82.5) | 89.4 (71.3, 112.2) | 69.8 (50.8, 95.8) |
| Oct-20 | 81.6 (77.5, 85.8) | 90.8 (72.9, 113.2) | 103.3 (75.5, 141.4) |
| Nov-20 | 92.9 (88.4, 97.6) | 103.8 (83.4, 129.3) | 88.1 (66.3, 117.2) |
| Dec-20 | 99.7 (95.0, 104.6) | 81.8 (65.1, 102.8) | 94.2 (70.9, 125.3) |
| Jan-21 | 89.7 (85.3, 94.2) | 91.8 (73.1, 115.2) | 87.2 (63.7, 119.6) |
| Feb-21 | 96.7 (92.0, 101.7) | 87.9 (69.4, 111.3) | 98.7 (72.6, 134.2) |
| Mar-21 | 92.3 (87.8, 97.0) | 97.6 (77.4, 123.2) | 114.4 (84.6, 154.7) |
| Apr-21 | 94.9 (90.5, 99.5) | 99.3 (79.2, 124.5) | 95.3 (70.8, 128.2) |
| May-21 | 99.0 (94.4, 103.7) | 119.4 (96.1, 148.3) | 125.1 (92.5, 169.1) |
| Jun-21 | 99.4 (94.8, 104.2) | 109.4 (88.3, 135.7) | 84.6 (62.4, 114.7) |
|  |  |  |  |
|  |  |  |  |
|  |  |  |  |
| **(c) Deprivation** | **Ratio of counts (95%CI) comparing 2021/20 to 2019 (%)** | | |
|  | **Least** | **Most** |  |
| Jan-20 | 92.7 (84.5, 101.8) | 102.7 (91.8, 114.8) |  |
| Feb-20 | 108.7 (98.9, 119.6) | 109.5 (97.7, 122.8) |  |
| Mar-20 | 121.5 (110.9, 133.1) | 122.7 (110.1, 136.8) |  |
| Apr-20 | 76.0 (69.0, 83.6) | 85.1 (75.8, 95.5) |  |
| May-20 | 53.1 (47.6, 59.2) | 48.4 (42.3, 55.5) |  |
| Jun-20 | 46.9 (41.9, 52.5) | 47.8 (41.8, 54.6) |  |
| Jul-20 | 65.3 (58.6, 72.7) | 61.2 (53.9, 69.4) |  |
| Aug-20 | 63.2 (56.8, 70.4) | 60.6 (53.5, 68.5) |  |
| Sep-20 | 77.5 (70.4, 85.4) | 81.8 (72.7, 92.1) |  |
| Oct-20 | 79.7 (72.3, 87.9) | 88.1 (78.3, 99.2) |  |
| Nov-20 | 91.9 (83.4, 101.2) | 91.2 (81.6, 102.0) |  |
| Dec-20 | 107.5 (97.9, 118.1) | 91.1 (81.4, 102.0) |  |
| Jan-21 | 92.0 (83.8, 101.0) | 88.1 (78.5, 99.0) |  |
| Feb-21 | 99.4 (90.2, 109.5) | 95.7 (85.0, 107.7) |  |
| Mar-21 | 93.7 (85.0, 103.2) | 99.4 (88.7, 111.4) |  |
| Apr-21 | 94.2 (86.1, 103.1) | 92.0 (82.1, 103.0) |  |
| May-21 | 97.8 (89.2, 107.1) | 106.3 (95.4, 118.5) |  |
| Jun-21 | 100.2 (91.6, 109.7) | 95.3 (85.5, 106.2) |  |
